# Disparities in spatial accessibility of primary care in Louisiana: From physical to virtual accessibility

**DOI:** 10.3389/fpubh.2023.1154574

**Published:** 2023-04-18

**Authors:** Fahui Wang, Yutian Zeng, Lingbo Liu, Tracy Onega

**Affiliations:** ^1^The Pinkie Gordon Lane Graduate School, Louisiana State University, Baton Rouge, LA, United States; ^2^Department of Geography and Anthropology, Louisiana State University, Baton Rouge, LA, United States; ^3^Department of Urban Planning, School of Urban Design, Wuhan University, Wuhan, China; ^4^Center for Geographic Analysis, Harvard University, Cambridge, MA, United States; ^5^Department of Population Health Sciences, University of Utah, Salt Lake City, UT, United States; ^6^Huntsman Cancer Institute, Salt Lake City, UT, United States

**Keywords:** two-step floating catchment area (2SFCA) method, two-step virtual catchment area (2SVCA) method, telehealth access, broadband availability, broadband affordability, primary care disparity, Louisiana

## Abstract

Telehealth has been widely employed and has transformed how healthcare is delivered in the United States as a result of COVID-19 pandemic. While telehealth is utilized and encouraged to reduce the cost and travel burden for access to healthcare, there are debates on whether telehealth can promote equity in healthcare services by narrowing the gap among diverse groups. Using the Two-Step Floating Catchment Area (2SFCA) and Two-Step Virtual Catchment Area (2SVCA) methods, this study compares the disparities of physical and virtual access to primary care physicians (PCPs) in Louisiana. Both physical and virtual access to PCPs exhibit similar spatial patterns with higher scores concentrated in urban areas, followed by low-density and rural areas. However, the two accessibility measures diverge where broadband availability and affordability come to play an important role. Residents in rural areas experience additive disadvantage of even more limited telehealth accessibility than physical accessibility due to lack of broadband service provision. Areas with greater Black population proportions tend to have better physical accessibility, but such an advantage is eradicated for telehealth accessibility because of lower broadband subscription rates in these neighborhoods. Both physical and virtual accessibility scores decline in neighborhoods with higher Area Deprivation Index (ADI) values, and the disparity is further widened for in virtual accessibility compared to than physical accessibility. The study also examines how factors such as urbanicity, Black population proportion, and ADI interact in their effects on disparities of the two accessibility measures.

## 1. Introduction

Equitable access to health services is an important concern in healthcare delivery and policy and a matter of social justice (1). The significance of healthcare accessibility research lies not only in treatment but also in prevention, and thus provides scientific decision support for the spatial allocation of medical resources (2). Access to primary care improves overall health and reduces disparities in health across major population subgroups (3). This paper examines disparities in spatial access to primary care *via* physical visit or telehealth.

Due to the impact of the COVID-19 pandemic, telehealth has been widely used to reduce face-to-face contact and has transformed healthcare delivery in the United States (4). The Health Resources and Services Administration (HRSA) of the U.S. Department of Health and Human Services (HHS) defines *telehealth* as “the use of electronic information and telecommunications technologies to support and promote long-distance clinical healthcare, patient and professional health-related education, and public health and health administration” (5). As early as 2013, the Louisiana Department of Health submitted a report to the House Committee on Health and Welfare and the Senate Committee on Health and Welfare on ways to expand telehealth services access in Louisiana (6). In 2021, in response to the COVID-19 emergency, the Louisiana Department of Health renewed the provider policy and managed care practices, which listed the range of telehealth and specified new requirements for providers to conduct telehealth (7).

Besides the advantages of reducing in-person contact during the pandemic, telehealth also decreases the stress associated with a hospital or clinic setting, especially in hospitalizations (6). Telehealth has been proven as an effective approach to save travel time and costs, especially for chronically ill patients, elders, females, and low-income residents with lower mobility and accessibility (8). For mental health services, telehealth increases feasibility and acceptability (9). As the utilization and satisfaction of telehealth increases during the pandemic (10, 11), telehealth is expected to be continually used post-pandemic (6). However, telehealth is not universally accessible. During the pandemic, people with lower socioeconomic status experienced worse health outcomes (12), with low access to telehealth as one factor in this disparity. The 2021 National Survey Trends in Telehealth Use found that telehealth utilization was lowest among the uninsured, individuals ages 18–24, Black individuals, and low-income respondents (13). Furthermore, with higher standards for reimbursable telehealth visits (e.g., the Louisiana Department of Health requires that providers and caregivers “must use interactive audiovisuals”), rural hospitals are least likely to be able to establish telehealth systems with patient engagement capabilities. Some rural hospitals cannot schedule appointments online, request refills, submit patient-generated data, view clinical records, or use online applications to access medical information (14).

In healthcare research applications, accessibility can be divided into spatial and non-spatial access (15). Spatial accessibility stresses the service providers (supply), residents (demand), and the geographic connection between them (16), while non-spatial accessibility captures how accessibility varies by characteristics of residents such as race, sex, income, family structure, educational attainment, homeownership status, and others (17). This research focuses on spatial accessibility, however, extends the analysis of disparities in spatial accessibility across geographic areas with different socio-demographic structures.

*Physical accessibility* refers to the relative convenience by which services can be reached *via* a physical visit from a given location. The earliest and perhaps most popular measure emphasizes *proximity*, e.g., minimum distance or travel time, to the closest service provider. Some use *cumulative opportunities* within a distance or travel time range to measure accessibility (18), and the *potential model* values supply at all locations, each of which is discounted by a distance decay effect (19). These may be termed as *supply-oriented accessibility measures* since they do not consider the amount of population competing for the service. In order to account for both supply and demand, a simple *supply–demand ratio method* computes the ratio of supply vs. demand in an area to measure accessibility. However, such a method cannot reveal detailed variations within the area unit nor consider supply–demand interaction between areas. The “*Two-Step Floating Catchment Area (2SFCA)*” method is developed to address these shortcomings (20). Its first step assigns an initial ratio in each service area centered at a supply location as a measure of supply availability (i.e., supply amount at that location divided by total demand within its catchment area). The second step sums up the initial ratios in the overlapped service areas to measure accessibility for a demand location, where residents have access to multiple supply locations. See section 3 for detailed formulation. The method considers interaction between demands and supply across areal unit borders and reveals the variation of accessibility within the area unit. Since its inception two decades ago, the 2SFCA method has been a popular measure of spatial accessibility. It overcomes the shortcomings of preceding methods that focus on either proximity to the nearest facility or simply supply–demand ratios within fixed geographical or administrative boundaries.

On virtual accessibility, this study introduces a method that refines an early version of “Two-Step Virtual Catchment Areas (2SVCA)” method (1). The conceptualization of virtual accessibility *via* telehealth still takes effect within a service provider’s physical catchment area since telehealth often works as supplementary consultation to reduce travel burdens for patients making physical visits (21). While the 2SFCA method captures the supply–demand interaction strength by a distance decay effect, the 2SVCA method models the virtual connection strength by the joint effect of digital transmission speeds at the supply and the demand locations. However, that pilot 2SVCA method focuses on the availability of quality internet service (e.g., broadband) in a geographic area but omits its affordability. In other words, not all residents can afford or have the technical know-how to take advantage of the available service. This study proposes a major refinement that separates the effects of broadband availability (whether the service is provided for residents or business in a geographic area) and affordability (whether and how many residents or business entities subscribe for the service) on telehealth access.

This study examines spatial accessibility of primary care physicians (PCPs) in Louisiana in two ways - namely “physical accessibility” *via* face-to-face interaction with care providers by the 2SFCA method, and “virtual accessibility” *via* telehealth by the *refined 2SVCA method*. While the body of health care access literature is rich on physical accessibility, its coverage on telehealth access, despite its increasing significance, remains largely at its infancy. The purposes of our study are three folded. First, it illustrates a novel method in data requirement and technical implementation. Secondly, results from the case study sheds light on how telehealth accessibility differs from traditionally physical accessibility, and whether telemedicine helps close the gap in access to health services (22), or exacerbate existing disparities (23). Thirdly, it helps inform the formulation of policy and planning strategies related to health care resource allocation, internet infrastructure as well as possible subsidy or financial assistance in promoting access more equitably.

## 2. Study area and data sources

The study area is Louisiana, comprised of 64 parishes with a total population of 4.66 million in 2020. “Parish” is the county equivalent unit in Louisiana. According to a recent report (24), Louisiana ranked the last among 50 states in the U.S. according to the Health Rankings Composite Measure in 2022. It highlights the importance of healthcare research, including primary care, in the study area. Primary care often serves as an entry point in the health care system.

**Figure 1:**
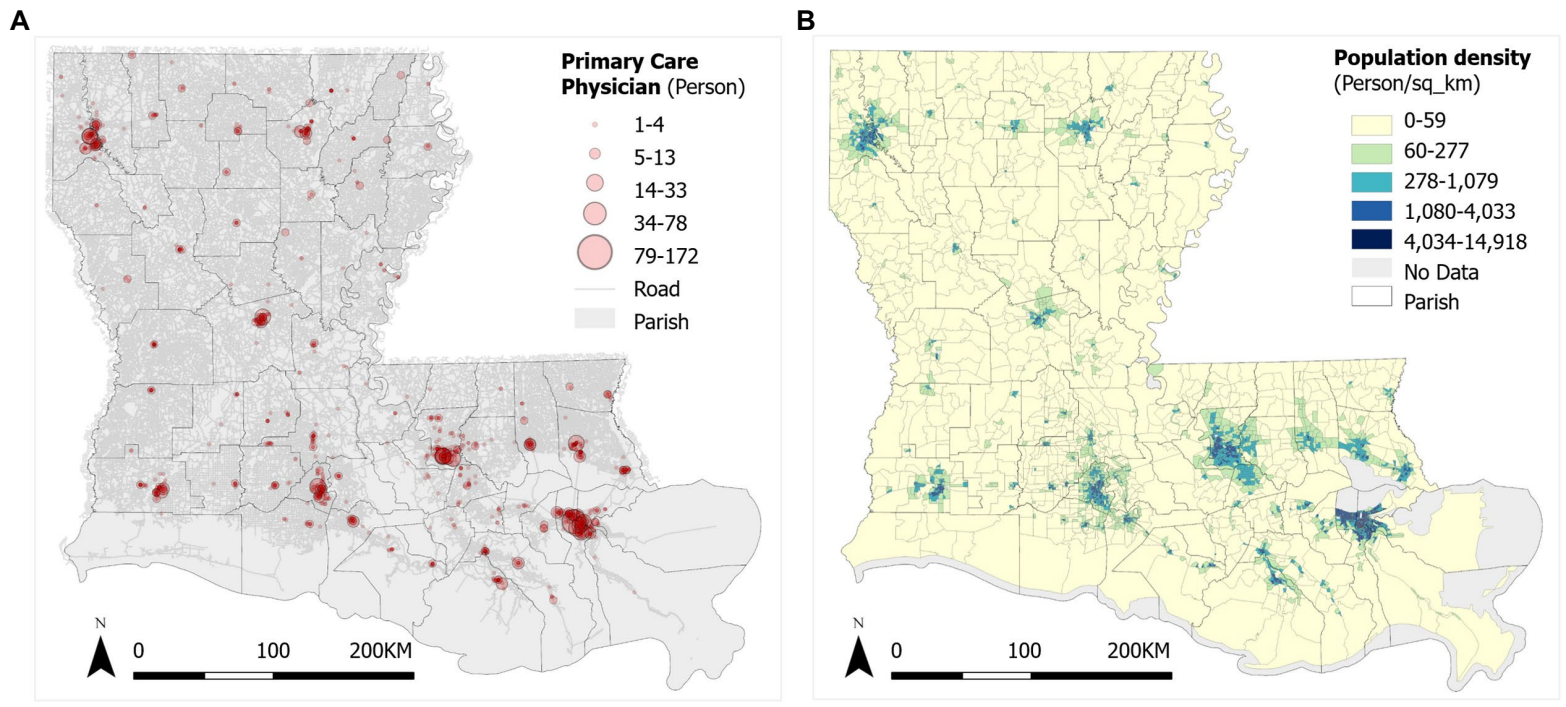
Distributions of **(A)** primary care physicians (PCPs) and **(B)** population density in Louisiana.

**Figure 2:**
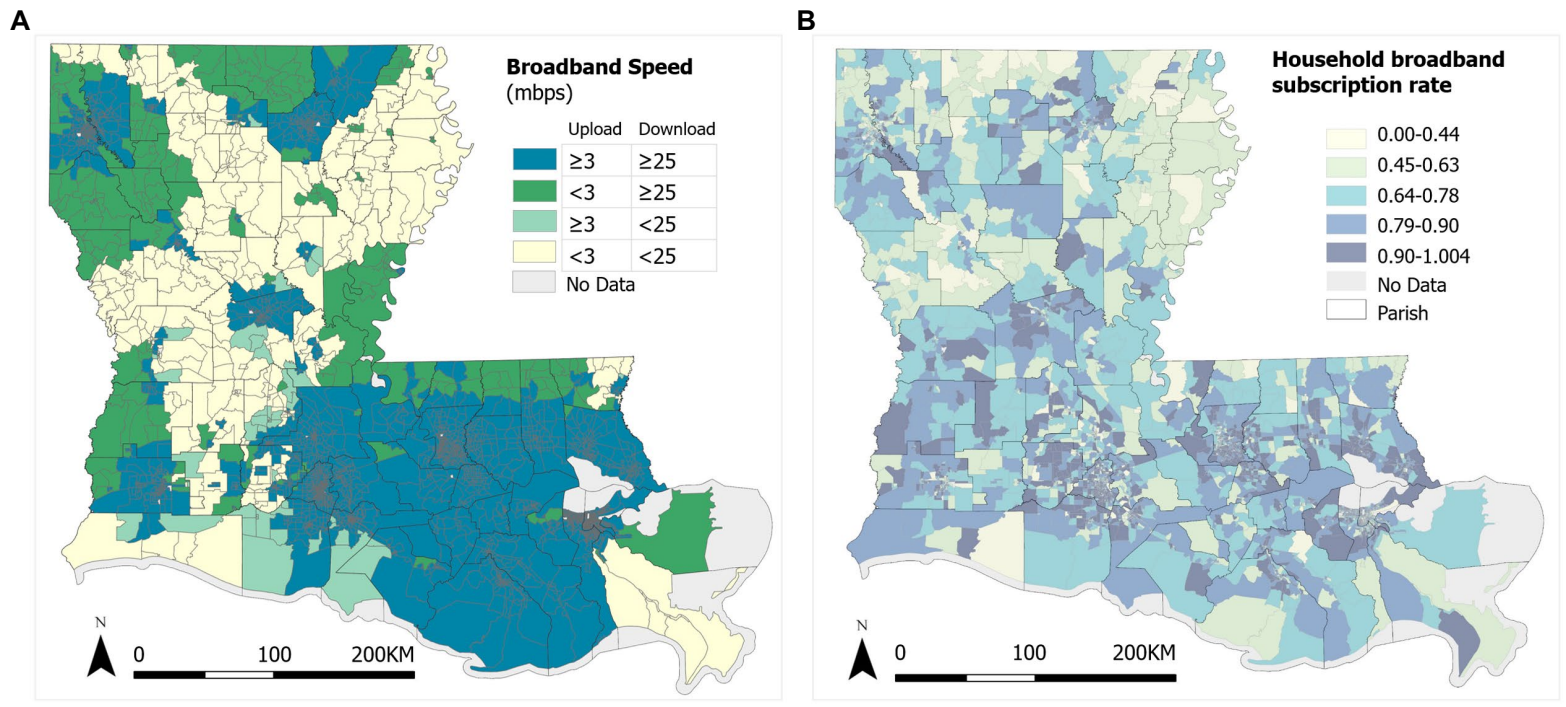
Broadband services in Louisiana: **(A)** Broadband download and upload speeds, and **(B)** subscription rates.

**Figure 3:**
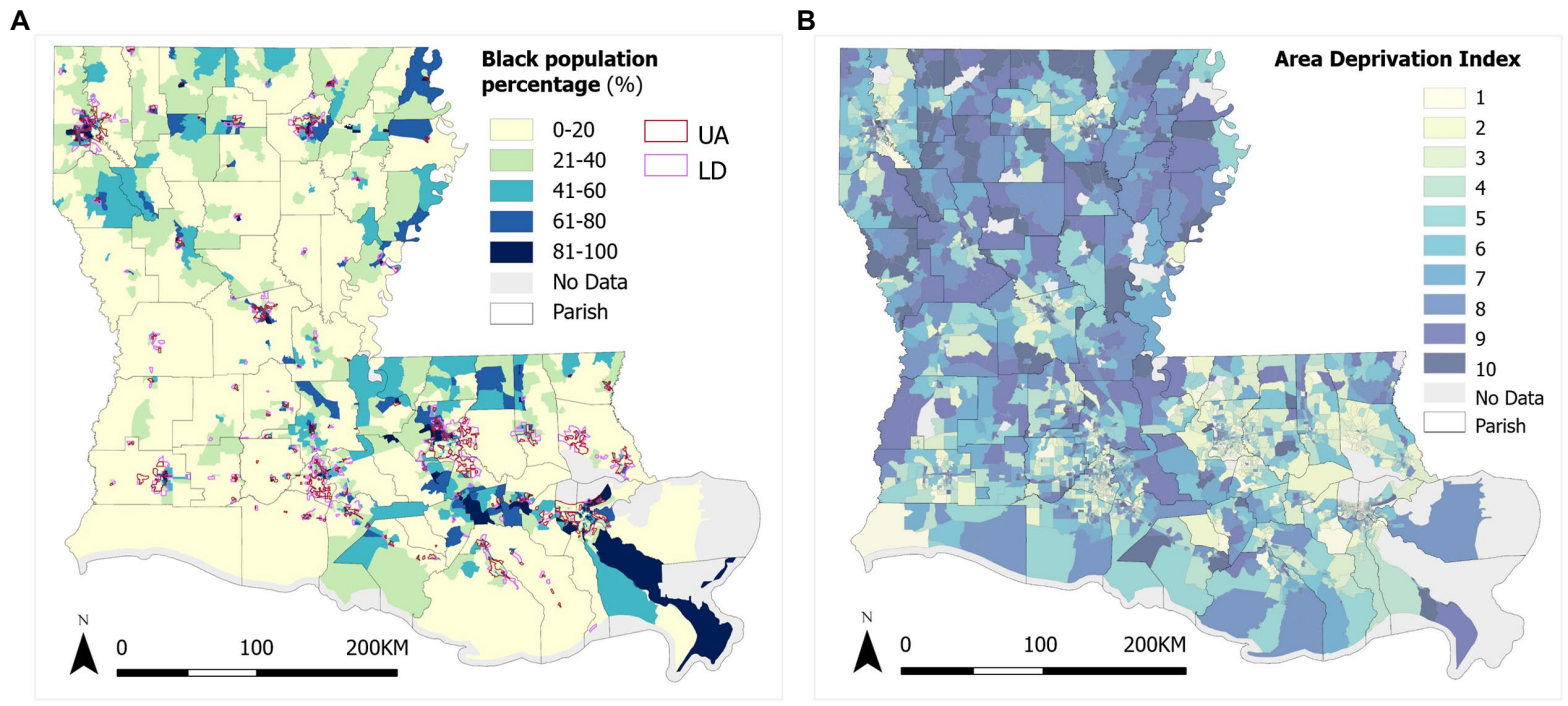
Variations of **(A)** Black population % and urbanicity, and **(B)** ADI in Louisiana.

Data sources for the study are composed of three parts. First, the variables needed for defining physical accessibility of PCPs include supply (physician facilities), demand (population), and road network that connects them. In addition to those three elements, the virtual accessibility measure needs internet data that help define broadband availability and affordability. Finally, examining the disparity of both accessibility measures is conducted across geographic areas of various urbanization levels (or urbanicity), concentration of minority population (e.g., Black individuals), and a consolidated index for concentrated disadvantages (i.e., ADI). The following details description of these data:

(1) *Data of Primary Care Physicians (PCPs) of Louisiana in 2022* come from the Doctors and Clinicians National Downloadable File released by the Centers for Medicare and Medicaid Services (CMS), in which full-time equivalent (FTE) is calculated as the service capacity at various locations provided by PCPs. There are 1,164 PCP locations and most are concentrated in urban areas and near the cities (Figure 1A). The *2020 Census Redistricting data* at the census block group level is utilized to define demand population in this study (25). Future work may adjust the demand based on health care needs by age, gender and other factors (26). In Louisiana, there are 4,294 block groups with population density ranging 0–14,918 persons per square kilometer (Figure 1B). *Road network data* with speed archives in Louisiana, downloaded from the Open Street Map data *via* Python OSMnx package (23), is used to calibrate the shortest drive time from each demand location (centroid of each census block group) to each supply location (PCPs). It produces a travel time matrix of 4,294 (block groups) × 1,164 (PCPs) = 4,998,216 O-D pairs. Due to the relative low ridership and limited coverage of public transit systems in the study area, this study does not consider travel time *via* transit.

(2) The *Federal Community Commission (FCC) Fixed Broadband Deployment Block Data* covers 2020 residential and business broadband data (27). The FCC data, released in 2010 census blocks, are transformed to 2020 census block groups in ArcGIS Pro. Specifically, the broadband download and upload speeds for each PCP location (supply) are the mean corresponding business broadband speeds for the 2010 block in which it is located, and the broadband speeds for population in each 2020 block group (demand) are the mean residential broadband speeds across 2010 blocks whose centroids fall within that 2020 block group. According to the FCC, it defines high-speed broadband as download speeds of up to 25 megabits per second and upload speeds of up to 3 megabits per second. A block group with broadband below this standard, simply denoted as 25/3 Mbps, is considered as an area without high-speed broadband availability. As shown in Figure 2A, the northwest part of Louisiana, especially those rural areas far from cities, are not covered by high-speed broadband. Data on households with an Internet subscription including broadband of any type is identified as B28002-004 from the *2016–2020 Five-Year American Community Survey (ACS)* (28). Figure 2B shows household broadband subscription ratios at the block group level in Louisiana in 2020, and the pattern is fragmented. In short, the broadband speeds from the FCC data along the threshold of 25/3 Mbps are used to define *broadband availability* as a binary parameter (i.e., 0 for being unavailable, and 1 for being available) for both PCP and residential locations, and the household broadband subscription ratios define *broadband affordability* in residential locations as a continuous parameter ranging 0–1.

(3) Based on the *2020 Census Urban and Rural Classification* with definition standard by housing units per square mile (HPSM), a census block group is defined as *urban area (UA)* (if density ≥ 425 HPSM), Low-Density Fill zone (hereafter simply referred to as *low density, LD*) (if density = 200 ~ 425 HPSM), and *rural area (RA)* (if density < 200 HPSM) (29). In Louisiana, 1,252 block groups are urban areas, 475 block groups are low-density fill zones, and 2,256 block groups are rural areas (Figure 3A). Thirty eight block groups are non-residential with negligible population (≤1) and thus excluded from the analysis. The *2020 Census Redistricting data* also have breakdowns by major racial groups. In Louisiana, there are 57.06% White population (non-Hispanic), 31.43% Black population (non-Hispanic), and the remaining 11.51% for others (American Indian or Alaska Native, Asian, and Native Hawaiian or Other Pacific Islander). Other racial-ethnic groups are not considered in analysis of racial-ethnic disparity because of their relatively low percentages. Also shown in Figure 3A, the concentrations of Black individuals tend to coincide with urban areas to some degree, but also in rural areas in the far north of the state as well as the northern edge on the east part of the state. Finally, the *Area Deprivation Index (ADI)* is considered a comprehensive metric that captures neighborhood socioeconomic disadvantage. ADI was based on a measure created by the Health Resources & Services Administration over three decades ago, and has since been refined, adapted, and validated to the census block group level (30). The index consolidates factors for the theoretical domains of income, education, employment, and housing quality, and has been frequently used to inform health delivery and policy, especially for the most disadvantaged neighborhood groups. The state ranking value of 2020 ADI has a range of 1 to 10 and is for each state alone without consideration of national levels (31). A higher ADI value corresponds to a more disadvantaged level (Figure 3B). High ADI values are observed in both local pockets in urban areas and prevalent in rural areas, especially in the northwest of the state. No ADI data are available for 67 block groups with low population or housing numbers.

Table 1 summarizes the basic statistics for some of the key variables across three urbanicity areas. In general, the Black population percentage increases from rural to low-density by about 10% and then to urban areas by another 10% on average. The average broadband subscription rate is the highest in low-density areas, drops to urban areas by 1.5%, and drops another 2.6% to rural areas. The lower subscription rate in urban areas is likely attributable to lower affordability in some low-income inner-city residents, and the lowest subscription rate in rural areas may be attributable to both lack of available broadband service providers there and poorer affordability for some of the residents. For both download and upload speeds, the urban advantage is evident, followed by low-density and then rural areas.

**Table 1:** Demography and broadband access by urbanicity in Louisiana.

	Population	Areas (km^2^)	White population (%)	Black population (%)	Broadband subscription (%)	Mean of max download (mbps)			Mean of max upload (mbps)		
						Min	Mean	Max	Min	Mean	Max
Total (*n* = 4,256)	4,652,718		55.74	33.43	77.53	13.55	161.38	1595.74	1.47	70.04	1,460.60
Rural area (*n* = 1,525)	1,629,554	121,857.67	69.86	21.52	75.69	13.55	84.50	910.94	1.47	17.03	815.32
Low density (*n* = 475)	579,612	2,173.06	58.51	31.14	79.78	16.37	165.54	1122.89	1.71	61.52	998.13
Urban area (*n* = 2,256)	2,443,552	2,333.97	45.61	41.95	78.29	13.56	212.47	1595.74	1.51	107.66	1,460.60

Urban area if density ≥ 425 HPSM, low density if density = 200 ~ 425 HPSM, and rural area if density < 200 HPSM.

## 3. The 2SFCA and 2SVCA methods

The *Two-Step Floating Catchment Area (2SFCA)* method is widely used in measuring spatial accessibility (32), and here, termed “physical accessibility” to emphasize the access *via* face-to-face visits to PCPs. The 2SFCA model for physical accessibility at demand location (census block group) *i* is written as:


(1) $$PA_{i}=\overset{n}{\underset{j\in \left(d_{ij}\leq d_{0}\right)}{\sum }}\left[S_{j}/\overset{m}{\underset{k\in \left(d_{kj}\leq d_{0}\right)}{\sum }}D_{k}\right]\text{,}$$

where supply capacity of PCPs at location *j* is denoted by *S_j_*, population at location *k* (or *i*) is denoted by *D_k_*, and the distance (here, drive time) between them is *d_kj_* (or *d_ij_*). The first step is for each supply (PCP) location *j*, search all demand locations (census block groups) *k* that are within a threshold drive time (*d_0_*) from location *j*, and compute the ratio of supply (number of PCPs) at *j* to the total demand (population) within that catchment area. The second step is for each demand location *i*, search all supply locations (*j*) that are within the threshold drive time (*d_0_*) from location *i*, and sum up the previously derived supply-to-demand ratios within its catchment area.

As a result, 2SFCA produces a ratio of supply to demands. Multiplying the ratio by 1,000 to avoid small numbers yields a number that can be interpreted as accessible PCPs per 1,000 residents. For simplicity, this study uses the conventional 2SFCA method in Equation (1) instead of the *generalized 2SFCA (G2SFCA)* (20). The latter accounts for the complexity of distance decay behaviors, which would require actual data of origin-to-destination (residents-to-PCP trip) flows to define a best-fitting distance decay function (33). For the catchment size *d_0_*, it is recommended that 30 min for primary care in the U.S. (21). However, travel time estimated in ArcGIS assumes free-flow travel speed and is likely to be underestimated. A prior study found an underestimation as much as about 5 min on average (34). Therefore, *d_0_* is set as 30–5 = 25 min.

Residents rarely use telehealth services from hospitals or physicians with which they do not have physical connections. Telehealth often works as supplementary consultation to reduce travel burdens for patients (35), and thus takes effect within a provider’s physical catchment area. Similar to the 2SFCA, the formulation of telehealth accessibility is also composed of two steps, each of which is confined to a virtual catchment area. Therefore, it is termed “*Two Step Virtual Catchment Area (2SVCA) method*” and measures “*virtual accessibility*” for residents (1). It was recently conceptualized to account for the availability and quality of internet services for both residents and service providers. This study further refines the 2SVCA by clarifying two distinctive elements that influence internet access: one is associated with a geographic area where quality internet (e.g., broadband service) may not be available, and another refers to the fact that even with available internet service provider(s) not all residents there can afford the service.

The refined 2SVCA method is formulated as:


(2) $$VA_{i}=a_{i}\overset{n}{\underset{j\in \left(d_{ij}\leq d_{0}\right)}{\sum }}\left[a_{j}b_{j}S_{j}/\overset{m}{\underset{k\in \left(d_{kj}\leq d_{0}\right)}{\sum }}\left(a_{k}b_{k}D_{k}\right)\right]\text{,}$$

where (1) either supply *S_j_* or demand *D_k_* participates in the virtual interaction between them depends on the broadband availability at their respective locations, denoted by *b_j_* and *b_k_*, and (2) only the portion (or whole) of *S_j_* or *D_k_* with the broadband subscription contributes to that interaction, denoted by *a_j_* or *a_k_*. Finally, the parameter *a_i_* (consumer broadband subscription rate) is applied to discount the initial virtual accessibility score assigned to demand location *i*, since only this fraction of residents has a consumer broadband subscription.

In this study, the broadband availability parameters *b_j_* and *b_k_* are associated with supply (PCPs) and demand (census block group) locations, respectively; and as stated previously, they are defined as binary 0–1 according to whether the internet (broadband) speeds exceed the threshold of 25/3 Mbps for business and consumers, respectively. The broadband affordability parameter at demand location, *a_i_* or *a_k_*, is represented by the household broadband subscription rate there; and this study assumes that broadband is affordable for all PCPs, thus their broadband subscription rates are uniform, i.e., *a_j_* = 1.

## 4. Disparities of physical and virtual accessibility by urbanicity, Black population proportion and ADI

The follow hypotheses are formulated to guide our case study:

1. Physical accessibility by the 2SFCA method is not different from virtual accessibility by the 2SVCA method;
2. Either accessibility measure does not differ significantly across areas of three urbanicity categories, various concentration levels of Black population, or area deprivation index (ADI) values; and
3. There are no interactions among urbanicity category, Black population concentration level and ADI index on their relationships with either accessibility measure.

This section examines the first two hypotheses, and section 5 examines the third hypothesis.

**Figure 4:**
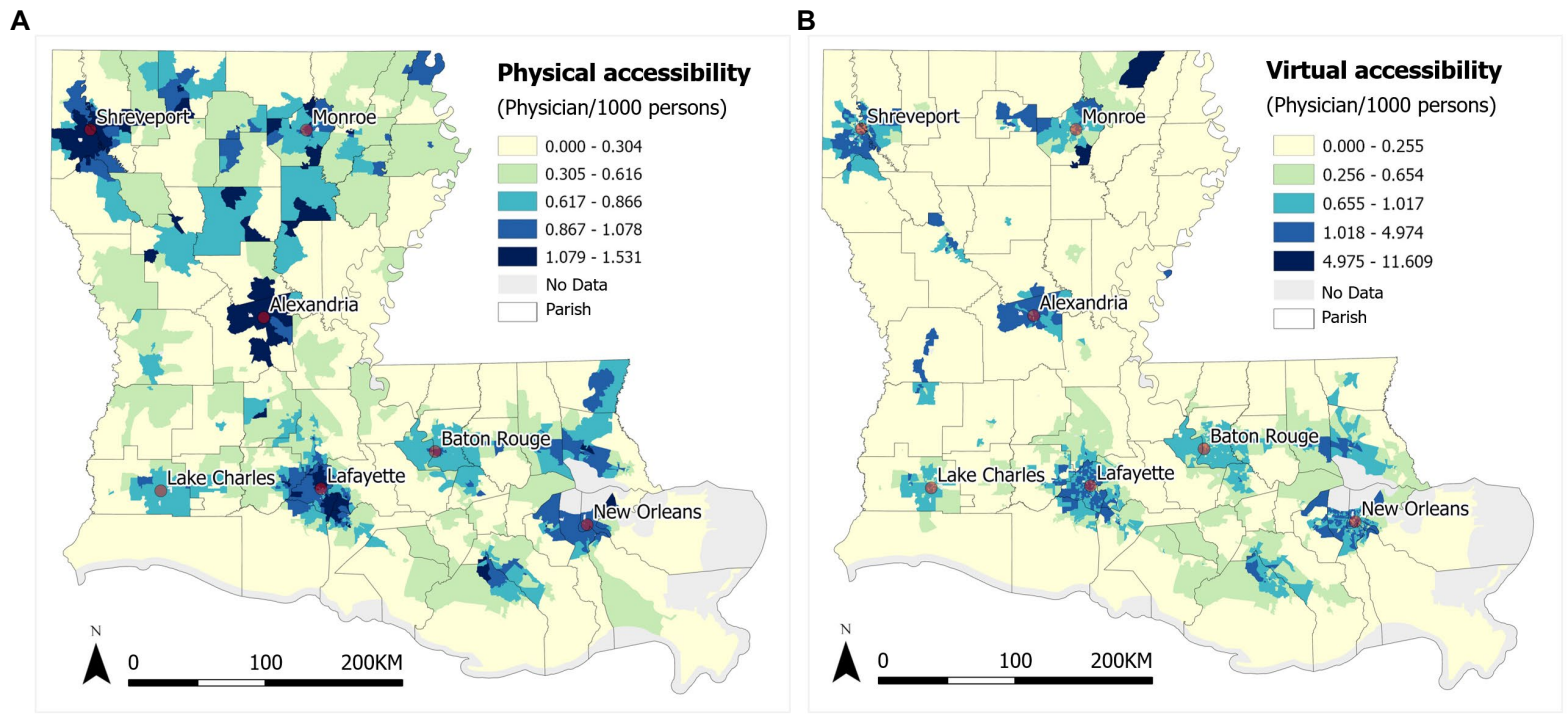
**(A)** 2SFCA physical accessibility and **(B)** 2SVCA virtual accessibility of PCPs in Louisiana.

**Figure 5:**
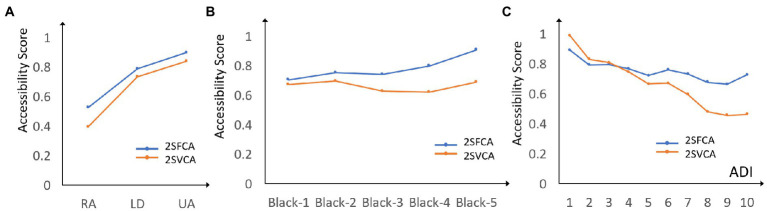
Mean accessibility scores of 2SFCA and 2SVCA across **(A)** urbanicity (RA, rural area; LD, low density; UA, urban area), **(B)** Black population proportion levels (1 = 0–20%, 2 = 20–40%, 3 = 40–60%, 4 = 60–80%, 5 = 80–100%), and **(C)** ADI.

Results of the accessibility scores obtained by the 2SFCA and 2SVCA methods are shown in Figures 4A,B, respectively, representing the number of physicians per 1,000 residents across block groups in Louisiana. In general, areas of higher accessibility are concentrated around cities and in the core areas of metropolitan areas, like Shreveport, Monroe, Alexandria, Lake Charles, Lafayette, Baton Rouge, and New Orleans, for both physical and virtual accessibility of PCPs. Due to long distances from PCPs and lack of high-speed broadband, rural areas fall behind in both measures (Figure 5A). The visual examination of the maps echoes a long tradition of examining the effect of urbanicity (i.e., degree of urbanization) on health behavior and outcome in health studies (36). Statistical analysis results reported in Table 2 confirm that the increases in both mean accessibility scores from rural to low-density and then urban areas are evident, and the differences are statistically significant.^1^

**Table 2:** Physical and virtual accessibility by urbanicity, Black population and ADI.

	No. block groups	2SFCA physical accessibility score					2SVCA virtual accessibility score				
		Mean	Median	Min	Max	Std. dev.	Mean	Median	Min	Max	Std. Dev.
	Total (*n* = 4,256)	0.756	0.825	0	1.531	0.338	0.672	0.747	0	11.608	0.488
Urbanicity	Rural area (*n* = 1,525)	0.529	0.496	0	1.503	0.362	0.398	0.238	0	11.608	0.581
	Low density (*n* = 475)	0.791	0.797	0	1.528	0.303	0.738	0.761	0	2.772	0.419
	Urban area (*n* = 2,256)	0.902	0.960	0.089	1.531	0.225	0.844	0.878	0	2.541	0.321
Black population	0–20% (*n* = 2002)	0.707	0.782	0	1.531	0.352	0.676	0.775	0	2.835	0.462
	20–40% (*n* = 804)	0.756	0.820	0	1.503	0.328	0.699	0.764	0	11.609	0.681
	40–60% (*n* = 488)	0.745	0.812	0	1.467	0.338	0.631	0.701	0	1.497	0.405
	60–80% (*n* = 411)	0.802	0.874	0	1.305	0.302	0.625	0.702	0	1.978	0.381
	80–100% (*n* = 551)	0.910	0.954	0	1.267	0.264	0.692	0.724	0	1.444	0.356
ADI	1 (*n* = 419)	0.895	0.960	0	1.273	0.205	0.993	1.045	0	2.130	0.272
	2 (*n* = 419)	0.797	0.872	0	1.447	0.307	0.834	0.897	0	1.661	0.379
	3 (*n* = 419)	0.799	0.877	0	1.358	0.319	0.812	0.877	0	2.772	0.403
	4 (*n* = 419)	0.770	0.812	0	1.531	0.310	0.750	0.805	0	1.818	0.392
	5 (*n* = 419)	0.726	0.809	0	1.467	0.369	0.670	0.760	0	4.974	0.481
	6 (*n* = 419)	0.764	0.841	0	1.467	0.333	0.675	0.720	0	2.835	0.433
	7 (*n* = 419)	0.735	0.820	0	1.445	0.359	0.597	0.674	0	1.638	0.406
	8 (*n* = 419)	0.679	0.738	0	1.528	0.356	0.485	0.514	0	1.397	0.394
	9 (*n* = 419)	0.667	0.692	0	1.503	0.376	0.459	0.416	0	9.600	0.610
	10 (*n* = 418)	0.730	0.777	0	1.291	0.353	0.466	0.421	0	11.609	0.672

Urbanicity includes urban area if density ≥ 425 HPSM, low density if density = 200 ~ 425 HPSM, and rural area if density < 200 HPSM; Black population (proportion in %); ADI, Area Deprivation Index.

There are also major differences between the two measures. The spatial pattern of physical accessibility in Figure 4A is steady and continuous with the highest scores in the urban cores and declining toward remote rural areas. As the spatial proximity to PCPs dominates the effect of 2SFCA method, much of the variability in the accessibility scores is smoothed out. The pattern of virtual accessibility in Figure 4B is rather sporadic with scattered low-score pockets in urban areas. This may be explained by the effect of low broadband subscription rates in urban poor in the 2SVCA method. The ranges of accessibility scores fall in a narrow range of 0–1.53 (standard deviation = 0.338) for 2SFCA but spread across a wide span of 0–11.608 (standard deviation = 0.488). In short, for the gap already experienced in physical accessibility between the urban and rural dwellers (33), the digital divide not just fails to close the gap, but even magnifies the disparity in telehealth access. Moreover, while the overall mean score of 2SVCA is lower than that of 2SFCA and consistent across the three types of urbanicity areas, the gap in the mean values between the two measures is the largest in rural areas, where the 2SVCA mean is 25% lower than the 2SFCA mean. This highlights the *triple challenges* in improving telehealth access in rural areas in Louisiana: farthest travel burden, least broadband service availability, and lowest affordability.

Our next task examines disparities in accessibility by concentration levels of a racial minority. For the reason stated previously, Black population is by far the largest minority group in Louisiana and thus chosen as an example. Since both accessibility scores and Black population proportion levels are area based (census block groups), not individuals, and the analysis has an ecological nature. The census block groups are classified into five levels with a 20% increment (Table 2). Our interest here is to assess whether Black individuals are disproportionally represented in areas of different levels of accessibility.

As shown in Table 2 and Figure 5B, as the Black population proportion level increases, the general trend is that the average 2SFCA scores increase with only a negligible dip from 20–40% to 40–60%, and the differences are all statistically significant (based on a similar regression as noted in footnote 1). In other words, when it comes to physical accessibility of PCPs, Black population tend to enjoy an advantage, or “reversed racial disadvantage” as previously reported (16). Such an advantage can be explained by higher Black population proportion levels in more urbanized areas in Louisiana (Table 1), where most PCPs are located.

For the 2SVCA (virtual accessibility or VA) scores, the regression result is reported below for clarity:


$$\begin{array}{l} VA=0.676+0.02\mathrm{3BLACK}2\_0.04\mathrm{5BLACK}3\\ \_0.05\mathrm{1BLACK4}+0.01\mathrm{6BLACK5} \end{array}$$

where the four dummy variables BLACK2-BLACK5 represent four levels of Black population % such as 20–40, 40–60, 60–80, and 80–100 while 0–20 is the reference category. None of the four coefficients is statistically significant at the 0.05 level. Also note that the coefficient signs alternate between positive and negative and thus indicate no clear trend of increasing or declining. That is to say, the average virtual accessibility scores have no statistically significant differences across the five Black population proportion levels. Figure 5B also supports this finding with no evident trend. In summary, the previously observed advantage in physical accessibility for Black population evaporates in virtual accessibility, most likely due to poorer affordability for high-quality internet experienced by a disproportionately high number of Black residents.

As shown in Table 2 and Figure 5C, the general trends are that both accessibility scores decline with increasing ADI values (i.e., higher levels of disadvantages), and the trend is more prominent for the 2SVCA-derived virtual accessibility than the 2SFCA-derived physical accessibility. The differences in either accessibility measure between the reference ADI category (i.e., ADI = 1) and any other ADI category are statistically significant. Both accessibility scores experience a minor uptick at the very end for ADI = 10. In other words, the disparity in virtual accessibility is further enlarged than the disparity in physical accessibility across the spectrum of ADIs. This is similar to the observation on the rural–urban gaps in the two accessibility measures (i.e., a larger gap in virtual than physical accessibility). For the more disadvantaged neighborhoods, residents not only endure poorer location in terms of PCP access *via* physical visit, and even much worse setting in telehealth access that is likely attributable to the same triple disadvantages outlined previously for rural residents—namely, poorest location (in terms of long distance from PCPs and/or few PCPs available within their range), least broadband service availability, and lowest affordability.

To recap, the existing disparities in physical accessibility of PCPs across both rural–urban and ADI spectrums are exacerbated in telehealth accessibility. The seeming advantage for physical access in neighborhoods of higher concentrations of Black individuals is eradicated for telehealth access.

## 5. Interactions of urbanicity, Black population proportion and ADI on accessibility measures

The previous section examines the variability of accessibility measures by each variable of urbanicity, Black population proportion and ADI. This section analyzes whether and how the interactions of these variables influence the accessibility scores. This is implemented by a 3-way ANOVA, and the result is summarized in Table 3.

**Table 3:** Three-way ANOVA on 2SFCA and 2SVCA scores.

	Df	2SFCA physical accessibility scores					2SVCA virtual accessibility scores				
		Sum Sq	Mean Sq	*F-*value	Prob (>F)	Estimate	Sum Sq	Mean Sq	*F-*value	Prob (>F)	Estimate
Intercept						0.552					0.572
Black population	1	21.1	21.09	261.46	2.0E-16***	−0.067	0.1	0.13	1.156	0.2824	0.085
Urbanicity	1	104.9	104.93	1300.848	2.0E-16***	0.142	206.8	206.83	1850.135	2.0E-16***	0.183
ADI	1	11.5	11.53	142.945	2.0E-16***	−0.038	76	75.99	679.803	2.0E-16***	−0.077
Black × Urbanicity	1	0	0.03	0.397	0.528	0.027	1	0.96	8.577	0.003**	−0.047
Black population × ADI	1	1.8	1.81	22.471	2.2E-06***	0.044	0.7	0.67	5.97	0.015*	0.029
Urbanicity × ADI	1	0.1	0.11	1.393	0.238	0.004	0.9	0.93	8.292	0.004**	0.009
Black population × Urbanicity × ADI	1	0.1	0.08	0.949	0.33	−0.007	0.1	0.07	0.666	0.414	−0.007
Residuals	4,171	336.5	0.08				466.3	0.11			

*Significant at 0.05, ** significant at 0.01, *** significant at 0.001. Df, Degree of freedom; ADI, Area Deprivation Index.

First of all, based on Table 3, both accessibility measures vary across the 3 urbanicity areas and across the 10 ADI categories, and the differences are highly statistically significant. The variation across Black population proportion levels is statistically significant for physical accessibility, but not virtual accessibility. The findings are consistent with those in the previous section, which are based on simple OLS regressions. However, the result from Table 3 provides stronger evidence for those findings as the 3-way ANOVA controls for the effect of interactions of the three variables.

**Figure 6:**
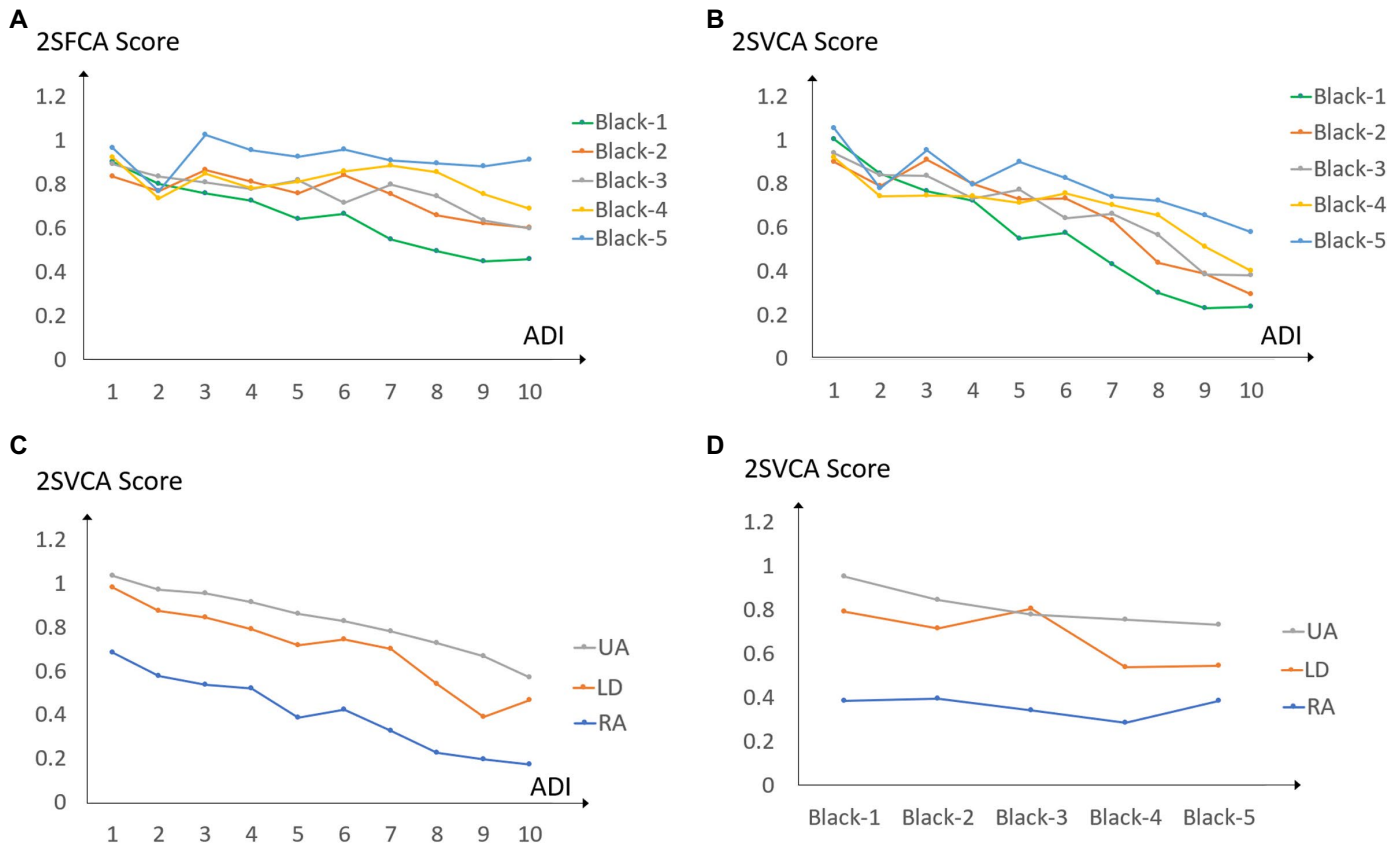
**(A)** 2SFCA scores by ADI and Black population; 2SVCA scores by **(B)** ADI and Black population, **(C)** ADI and urbanicity, **(D)** Black population and urbanicity.

The joint interaction of all three variables is not significant for either accessibility measures. Therefore, our discussion focuses on the effects of two-variable interactions. In other words, the 3-way ANOVA is downscaled to multiple 2-way ANOVAs. On the physical accessibility scores, only the interaction of Black population proportion and ADI is significant; and on the virtual accessibility scores, all 3 two-variable combinations exert significant effects. In Figures 6, 4 graphs are presented to show the corresponding four significant effects: (a) for the Black population-ADI joint effect on 2SFCA scores, and (b)–(d) for three joint effects on 2SVCA scores by Black population-ADI, urbanicity-ADI, and urbanicity-Black population. Each data point in any graph of Figure 6 represents the average accessibility score for an intersected sub-group. For example, the data point at the very end of the blue line (upper top, labeled Black5) in Figure 6A corresponds to the average 2SFCA score for census block groups with 80–100% Black population and ADI = 10.

In Figure 6A, when the ADI values are lower (1, 2), the relationship between the 2SFCA scores and Black population proportion levels is not definitive; when ADI ≥ 3, the pattern is largely consistent as higher Black population proportion levels generally correspond to higher 2SFCA scores (though the difference between variables Black2 and Black3 is ambiguous as the two lines cross each other); when ADI ≥ 6 for the most disadvantaged neighborhoods, the downward lines indicate declining physical accessibility with increasing ADI. In other words, the positive correlation of Black population proportions and 2SFCA scores is more evident in areas with moderate and high ADI values, but higher ADI values are associated with lower 2SFCA scores in areas with the highest concentrated disadvantages. The findings on the overall trend between the 2SFCA accessibility measure and Black population proportion (or ADI) derived in the previous section only apply to specific ranges of ADI categories. This revelation would not be feasible without the ANOVA.

In Figure 6B, the 2SVCA scores decline with increasing ADI values consistently across almost all Black population proportion levels, and the declining slope is the steepest for areas with the lowest Black population proportion (0–20%). That is to say, a higher ADI is a driving force for lowering 2SVCA scores, and such an effect is most prominent in neighborhoods dominated by White individuals. Given the same ADI value, especially in disadvantaged neighborhoods with ADI ≥ 5, higher Black population proportion levels correspond to higher 2SVCA scores. Our statistical analysis reveals that such an observation is statistically significant (after controlling for the effect of ADI). The underlying forces are the overlapping effects of better broadband availability and lower broadband subscription rates in areas of higher Black population proportion levels, and the former is stronger to offset the latter and leads to better 2SVCA scores.^2^

In Figure 6C, once again, higher ADIs are associated with poorer telehealth accessibility, and such a trend is largely consistent across the rural–urban spectrum. In Figure 6D, higher Black population proportion levels tend to be associated with lower 2SVCA scores in urban areas, to a less degree in low-density areas, and not at all in rural areas. Recall the finding from the previous section that no statistically significant relationship is found between Black population proportion levels and 2SVCA scores. That is likely to be caused by the divergent trends of their correlation across urbanicity areas. Once again, the ANOVA reveals a previously undetectable relationship between higher Black population proportion and lower 2SVCA scores in a specific geographic setting (urban areas), and our statistical analysis confirms that such a relationship is significant.^3^

To recap the results from ANOVA, we emphasize the findings not revealed from the previous section. The positive association between 2SFCA scores with Black population proportion levels and the negative association between 2SFCA scores with ADI values are most evident in areas in upper ADI ranges. For the 2SVCA virtual accessibility, a higher ADI is a consistent force driving its value down, and such an effect is most pronounced in areas of lower Black population percentage. In addition, the negative association between Black population proportion and 2SVCA score is mostly an urban phenomenon.

## 6. Concluding comments

This study examines the spatial accessibility of primary care *via* face-to-face visit and telehealth, and termed physical and virtual accessibility, respectively. The former is implemented by the conventional 2SFCA method, and the latter is by the newly formulated 2SVCA method. The 2SVCA method is conceptualized on the basis of 2SFCA as most telehealth happens between patients and service providers that are already connected *via* physical visits. Within the existing physical catchment areas, the 2SVCA method adjusts accessibility by imposing additional constraints related to internet service. One constraint is termed “broadband availability parameter” to reflect whether quality internet such as broadband is provided in a geographic area, and another constraint is termed “broadband affordability parameter” to capture the portion of residents with subscription to broadband. The difference between the two accessibility measures is a joint effect of the two parameters.

The case study in Louisiana focuses on the disparity of both accessibility measures across areas of various urbanization levels, areas with different concentrations of racial minority such as Black individuals, and areas with varying ADI values. Overall, the two measures have consistent patterns such as increasing access from rural to low-density and to urban areas, and declining access from low-ADI to high-ADI areas. In both cases, the disparities across urbanicity types and ADI spectrum are enlarged for the virtual accessibility. Higher Black population proportion levels tend to be associated with better physical accessibility, but such an advantage is not materialized in virtual accessibility. Our analysis on the effect of interactions among the factors of urbanicity, Black population proportion and ADI reveal more details in their relationships with the accessibility measures. Those overall trends identified from the full data set are more pronounced in some areas than others. In short, the existing disparities in physical access to primary care are exacerbated in telehealth access for the have-nots in areas such as rural and with concentrated disadvantages. The seemingly locational advantage in physical access for Black population concentrated neighborhoods becomes nonexistent in telehealth access.

Some major lessons can be learned. Telehealth accessibility is driven by more forces and thus more complex than physical accessibility. It adds the interaction of internet availability and affordability to physical accessibility that is dictated by where the PCPs and residents are and the transportation network(s) that connect them. For telehealth to make a difference in narrowing health care disparity, one looks no further than widening the broadband service provision to currently uncovered space and bringing down the financial and cultural barriers (34) for high quality internet service (e.g., subsidy for targeted population, provision of devices, fostering trust). One may consider mobile communication *via* cellular as a reasonable mode of remote health care in low-resource settings (35). The current conceptualization of virtual accessibility is at a pilot stage, and its formulation largely relies on the belief that telehealth is contingent upon (or supplementary to) regular visits to service providers. It calls for further refinements in light of future analyses of internet user experience data and telehealth utilization data.

## Data availability statement

The original contributions presented in the study are included in the article/supplementary material, further inquiries can be directed to the corresponding author.

## Author contributions

FW designed the study, directed its implementation, revised the manuscript, and gave final approval for the submission. YZ and LL implemented the study and drafted the manuscript. TO co-led in conceptual development with FW and provided manuscript and scientific review. All authors read and approved the manuscript and participated sufficiently in, and stand by the validity of this work.

## Funding

This work was supported by the National Cancer Institute (grant no. R01CA267990–01).

## Conflict of interest

The authors declare that the research was conducted in the absence of any commercial or financial relationships that could be construed as a potential conflict of interest.

## Publisher’s note

All claims expressed in this article are solely those of the authors and do not necessarily represent those of their affiliated organizations, or those of the publisher, the editors and the reviewers. Any product that may be evaluated in this article, or claim that may be made by its manufacturer, is not guaranteed or endorsed by the publisher.

## Footnotes

^1^This is based on a simple OLS regression with the accessibility scores as y and two dummy variables x1 and x2 to code the 3 urbanicity types, e.g., assuming values 0, 0 for "rural" as reference category, then 1, 0 for "low-density," and 0, 1 for "urban." In the regression results, the coefficients for x1 and X2 represent the differences of (1) "low-density" vs. "rural," and (2) "urban" vs. "rural," respectively; and the corresponding t-values indicate whether the differences are statistically significant.

^2^Using a subset of data for ADI ≥ 5, a regression of 4 dummy variables for Black population proportion levels and 1 additional explanatory variable ADI (to control for its effect) on broadband availability (parameter *b*) indicates that the coefficients increase with higher Black population proportion levels and are all statistically significant; another regression of 4 dummy variables for Black population proportion levels and 1 additional explanatory variable ADI (to control for its effect) on broadband subscription rates (parameter *a*) indicates that the coefficients decreases with higher Black population proportion levels and are all statistically significant.

^3^Using a subset of data for urban areas, a regression of 4 dummy variables for Black population proportion levels on 2SVCA scores indicates that the coefficients decrease with higher Black population proportion levels and are all statistically significant.
